# Incorporation of the nanoencapsulated polyphenolic extract of *Ferula persica* into soybean oil: Assessment of oil oxidative stability

**DOI:** 10.1002/fsn3.1575

**Published:** 2020-04-19

**Authors:** Parviz Estakhr, Javad Tavakoli, Faranak Beigmohammadi, Shima Alaei, Amin Mousavi Khaneghah

**Affiliations:** ^1^ Department of Food Science and Technology Faculty of Agriculture, Kermanshah Branch Islamic Azad University Kermanshah Iran; ^2^ Department of Food Science and Technology Faculty of Agriculture Jahrom University Jahrom Iran; ^3^ Department of Agronomy and Plant Breeding Faculty of Agriculture, Kermanshah Branch Islamic Azad University Kermanshah Iran; ^4^ Department of Food Science Faculty of Food Engineering University of Campinas (UNICAMP) Campinas Brazil

**Keywords:** antioxidant effect, coating materials, Ferula persica extract, nanoencapsulation, oxidative stability, polyphenols

## Abstract

In the present study, for the first time, the biological activities of *Ferula persica* extract (FPE) coated with locust bean gum (LBG) and chitosan in W/O/W emulsions were investigated. Based on the findings, the Z‐average size of emulsions coated by chitosan, LBG, and the complex of chitosan and LBG (1:1) (CCL) was 115.47, 128.37, and 68.12 nm, respectively. The encapsulation efficiency of the phenolic extracts in the powder produced by chitosan, LBG, and CCL decreased from 85.3 to 64.1, from 89 to 71.4, and from 93.3% to 77.9% during 24‐day storage, respectively. Also, the application of the coating in the encapsulation of FPE increased the antioxidant efficacy in soybean oil while compared with tert‐butylhydroquinone (TBHQ) and un‐encapsulated FPE. In this regard, The FPE nanoencapsulated by CCL showed the best antioxidative activity in soybean oil, followed by the FPE of nanoencapsulated by LBG and chitosan, respectively, which can be correlated with higher levels of polyphenolic compounds release over time in the sample coated with CCL. In this context, the encapsulation with CCL can be proposed as a promising technique to improve the antioxidant activity of extracts.

## Graphical abstract

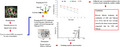

## 1 INTRODUCTION

The application of antioxidants in foods to prevent the activity of free radicals is essential which can cause further increases in their shelf life (Akbarirad, Gohari Ardabili, Kazemeini, & Mousavi Khaneghah, 2016). While due to adverse health effects of synthetic antioxidants, their uses are declining, the application of natural antioxidants is expanding (Carneiro, Tonon, Grosso, & Hubinger, 2013; Carneiro et al., 2013; Hashemi et al., 2018). In this regard, the investigation regarding the antioxidant activity of natural compounds attracted notable attention (Delfanian, Razavi, Haddad Khodaparast, Esmaeilzadeh Kenari, & Golmohammadzadeh, 2018; Mohammadi, Jafari, Esfanjani, & Akhavan, 2015; Rajaei, Barzegar, Mobarez, Sahari, & Esfahani, 2010). The application of herbal extracts and essential oils in the stabilization of food products such as edible oils has been carried out in a variety of studies, which in most of these cases has had a positive effect (Es, Mousavi Khaneghah, & Akbariirad, 2017; Hashemi, Mousavi Khaneghah, Khoshnoudinia, Mahdavian Mehr, & Khuzistani, 2013; Hashemi, Mousavi Khaneghah, Souza, & Sant'Ana, 2017; Lorenzo et al., 2018). However, the effectiveness of natural antioxidants while are added to food products is a matter of concern.

In the early stages of edible oils storage, in order to prevent the oxidation reactions, only a small amount of antioxidants is needed (Delfanian et al., 2018; Mohammadi, Jafari, Assadpour, & Esfanjani, 2016; Mohammadi et al., 2015) while after this stage, the progressive incorporation of antioxidants is the best strategy to keep their efficiency. In this regard, the encapsulation technique is used to keep and improve the functional properties of bioactive compounds including antimicrobial, nutritional value and antioxidant activities (Bhattia, Khalid, Uemuraa, Nakajima, & Kobayashi, 2017; Mohammadi et al., 2015; Uchiyama, Chae, Kadota, & Tozuka, 2019). In this method as a widespread technology in the food and pharmaceutical industry, the bioactive compounds are packed using a coating material such as macromolecules, before their incorporations into food products. While the main goal of this process is the gradual release of these compounds, however, the further impartments in the keeping of encapsulated bioactive compounds are among other achievements by this technique (Ezhilarasi, Karthik, Chhanwal, & Anandharamakrishnan, 2013; O’Regan & Mulvihill, 2009).

Among the common approaches of the above‐mentioned technique, the transformation of plant extracts into nanoemulsion systems can be considered as one of the most promising applications of encapsulation (Mohammadi et al., 2015; Mozafari et al., 2006). When an emulsion contains droplets with a mean diameter greater than 200 nm, it is usually categorized as a conventional emulsion. But when it contains droplets with a mean diameter smaller than this value can be referred as a nanoemulsion. However, there is currently no consensus on the upper limit for the mean droplet diameter defining a nanoemulsion (10 to 1,000 nm) (Choi & McClement, 2020). Therefore, nanoencapsulation can improve and modify the bioavailability and controlled release of compounds while compared with microencapsulation (microparticles are between 3 and 800 μm) (Delfanian et al., 2018; Iranshahi, Amin, Amini, & Shafiee, 2003). However, in this approach, the release of materials into their outer environment is influenced by the size of the particles. In this context, according to Mohammadi et al. (2016), the concentration of phenolic compounds released from nanoencapsulated olive leaf extract by concentrated whey protein during 20 days at 30°C was less controlled while compared with those encapsulated with the combination of concentrated whey protein and pectin which can be correlated with role of pectin in reducing the release rate of phenolic compounds. Based on the findings of Delfanian et al. (2018), the increase in oxidative stability of soybean oil by the incorporation of the nanoencapsulated phenolic extract of *Pistacia atlantica* hull oil was higher than those obtained by the incorporation of free extract.

*Ferula persica,* belongs to the Apiaceae family, is one of the plants that grow in different parts of Iran mainly Semnan province (Javidnia, Miri, Kamalinejad, & Edraki, 2005). Due to antioxidative activity and antimicrobial activities, as well as a high content of compounds such as alkaloids, carotenoids, and flavonoids, the application of this plant beside the derived extracts attracted notable attention (Dehpour, Ebrahimzadeh, Fazel, & Nabavi, 2009). Moreover, the antioxidative effect of *F. asafetida* extract as a good chelating agent was demonstrated (Dehpour et al., 2009).

In recent years, natural hydrocolloids have been increasingly used in the food industry to improve stability, functional properties, quality and safety, and nutritional and health benefits of different food products such as beverages, bakery and confectionary, sauces and dressings, meat and poultry products (Yemenicioglu, Farris, Turkyilmaz, & Gulec, 2019). One of the most commonly used types of gum in the food industry is locust bean gum which is extracted from seed of locust tress as extensively grown plant in Spain and other Mediterranean countries (Dakia, Blecker, Robert, Wathelet, & Paquot, 2008). Also, chitosan a linear polysaccharide consisting of (1,4)‐linked 2‐amino‐deoxy‐b‐d‐glucan, is a deacetylated derivative of chitin, which is the second most abundant polysaccharide found in nature after cellulose (Aider, 2010).

Therefore, for the first time, the *Ferula persica* extract was subjected to the nanocapsulation process by the aid of the locust bean gum, chitosan, and a combination of chitosan and locust bean gum (1:1). Then the prepared nanocapsulated extracts were incorporated into soybean oil samples, and furthermore, the oxidative stability of was investigated.

## 2 MATERIALS AND METHODS

### 2.1 Materials

Twenty kg of *F. persica* plant was collected from Semnan (Summer of 2018), and after immediate packing with nitrogen, it was transformed to Shiraz by airplane. Locust bean seeds and chitosan were purchased from the Tabibdaru and Sigma Companies, respectively. Soybean and sunflower oils with no antioxidants were also provided from Shiraz Narges edible oil Company, Shiraz, Iran.

### 2.2 Extraction Process

After manually cleaning, the *F. persica* plant was dried (it was exposed to sunlight for 36 hr.) and then completely powdered by the aid of a grinder (Mullinex Depose‐Brevete S.G.C.G.). Afterward, 100 g of dried powder was mixed with 1 liter of ethanol–water solvent (53.5:46.5) and the sample‐containing Erlens were then sonicated in an ultrasonic bath (DT 102H; BANDELIN) (35 kHz) for 34.1 min at 52.9°C (Carneiro et al., 2013; Carneiro et al., 2013; Hashemi et al., 2018).

### 2.3 Total phenolic content

In order to measure the total phenolic content of different extracts and oil samples, a previously described method was used (Tavakoli et al., 2018; Tavakoli, Sedaghat, & Mousavi Khaneghah, 2019a).

### 2.4 Extraction of locust bean gum

Locust bean gum (LBG) was prepared according to a previously described method (Dakia et al., 2008).

### 2.5 Biopolymer solution preparation

LBG, chitosan, and complex of chitosan and LBG (1:1 ratio) (CCL) were used as a wall‐covering material. LBG was mixed in deionized water to achieve a total solids content of 0.5% (w/w). A magnetic mixer was used to the better dissolution of the compounds for 15 min at 20°C, and then the solutions were kept in the refrigerator for 24 hr. Also, 0.5 g of chitosan was dissolved in 1,000 ml of 2% acetic acid and stirred for 30 min. Then, this solution centrifuged at 9,520 *g* at 20°C. The complex solutions of chitosan and LBG were prepared by adding an LBG solution into the solution of chitosan and stirring at 20 Cº.

### 2.6 W/O/W double emulsions preparation

The W/O/W two‐layer nanoemulsions were prepared using two emulsion‐forming steps. First of all, W/O micro‐emulsion was prepared by dropwise addition of 7% *F. persica* extract in a continuous phase containing 25% span 80 and 68% soybean oil without antioxidant. In the second phase of emulsification, W/O initial micro‐emulsion was coated with biopolymers prepared to produce W/O/W double emulsions. As a result, 30% initial W/O emulsion was added to 70% prepared bipolymers and homogenized at 10°C for 5 min at 13,709 *g* (10,161 *g*) and then at 30,845 *g* (15,972 *g*) for 8 min. Then, the homogenizer (Avestin EmulsiFlex C3, ATA, USA) was used at a pressure of 9,000 to 13,000 psi in 3, 5, and 7 cycles (90 s each) to reduce the particle size and to better stabilize the emulsion (Delfanian et al., 2018; Mohammadi et al., 2015).

### 2.7 Properties of emulsion

#### 2.7.1 Particles size distribution

The mean particle diameter (Z‐average), particle size distribution, and polydispersity index (PDI) were determined based on the recommended technique by Delfanian et al. (2018) using the dynamic light scattering (DLS) instrument (Zetasizer Nano ZS, Malvern Instruments, Malvern, England). In this regard, the samples were diluted 100‐fold with deionized water to avoid multiple scattering and measurements were carried out at 25°C.

#### 2.7.2 ζ‐potential

The **ζ‐**potential of emulsions was measured based on their electrophoretic mobility by a combination of laser Doppler velocimetry and phase analysis light scattering technique (Zetasizer Nano ZS, Malvern Instrument, England). In this context, the samples were diluted in deionized water at a ratio of 1:100 (v/v) to avoid multiple scattering (Delfanian et al., 2018; Mohammadi et al., 2015).

#### 2.7.3 Freeze‐drying nanoemulsions

Prepared nanoemulsions were frozen for 24 hr at −50°C and then lyophilized in a freeze dryer (Martin Christ, 8891, type 317, Germany) at *P* = 0.09 mbar and *T* = 0.01°C for 48 hr. The freeze‐dried encapsulated samples were converted into powder with help of a pestle and mortar (Delfanian et al., 2018).

### 2.8 Analysis of powders

#### 2.8.1 Encapsulation efficiency

For this purpose, 0.5 g of nanoencapsulated powders was mixed with 2 ml of ethanol–methanol (1:1) and vortexed for 2 min. The resulting mixture was then straightened with filter paper (No. 1). The amount of phenolic compounds was determined based on the Folin–Ciocalteau method. Finally, the encapsulation efficiency was determined based on the following formula:

$$\text{EE}\left(\%\right)=100-\left(P_{2}/P_{1}\times 100\right)$$

where *P*_2_ is the surface of phenolic compounds and *P*_1_ is theoretical total polyphenol content (Robert et al., 2010).

#### 2.8.2 Evaluating the release properties

The stability of encapsulated extracts was determined based on the release rate of phenolic compounds present in the inner part of W/O/W nanoemulsion. Approximately 12 g of nano‐sized samples were poured into dark glass containers and were placed in an oven at 30°C for 24 days. At the end of each 4 days, the amount of phenolic compounds was determined according to the method described in Section 2.3 (Delfanian et al., 2018).

The rate constant (k) and half‐life period (t_1/2_) of encapsulated powders were determined from the slope of the semi‐logarithm plotted of their remaining contents in nanocapsules versus storage time. The half‐life of polyphenols “*t*_1/2_^”^, which is defined as the time of a reduction of 50% of their initial values in the capsules, was calculated from the slope of the curve and based on the *t*_1/2_ = 0.693/k (Chranioti, Nikoloudaki, & Tzia, 2015).

### 2.9 Peroxide value and p‐anisidine value

The measurement of peroxide value and p‐anisidine value of the different oil samples used in the present study was done according to the method described by Tavakoli, Hajpour Soq, et al. (2019) and Delfanian et al. (2018), respectively.

### 2.10 Statistical analysis

In the present study, all experiments were performed in three replications and the results were analyzed by analysis of variance (ANOVA) (MStatC). Also, Slide Write and Excel software were used to prepare regression and graphs, respectively. Duncan's test was used to compare the mean values.

## 3 RESULTS AND DISCUSSION

### 3.1 Distribution and size of droplets of emulsions and ζ‐potential

The distribution of droplet size and PDI of multi‐emulsions with chitosan, LBG, and CCL were measured using the DLS method under diluted conditions. One of the most important and most consistent parameters set by DLS is the Z‐average size (Bryła, Lewandowicz, & Juzwa, 2015). However, several different high‐energy emulsification devices were used in food science, the most used emulsification processes are high‐pressure homogenizer and Rotor–Stator Mixer, while the high‐pressure homogenizer is the standard tool for emulsification of low‐to‐intermediate viscosity dispersions, and the Rotor–Stator Mixer is the standard tool for dispersions with higher viscosities (Håkansson, 2019).

**FIGURE 1:**
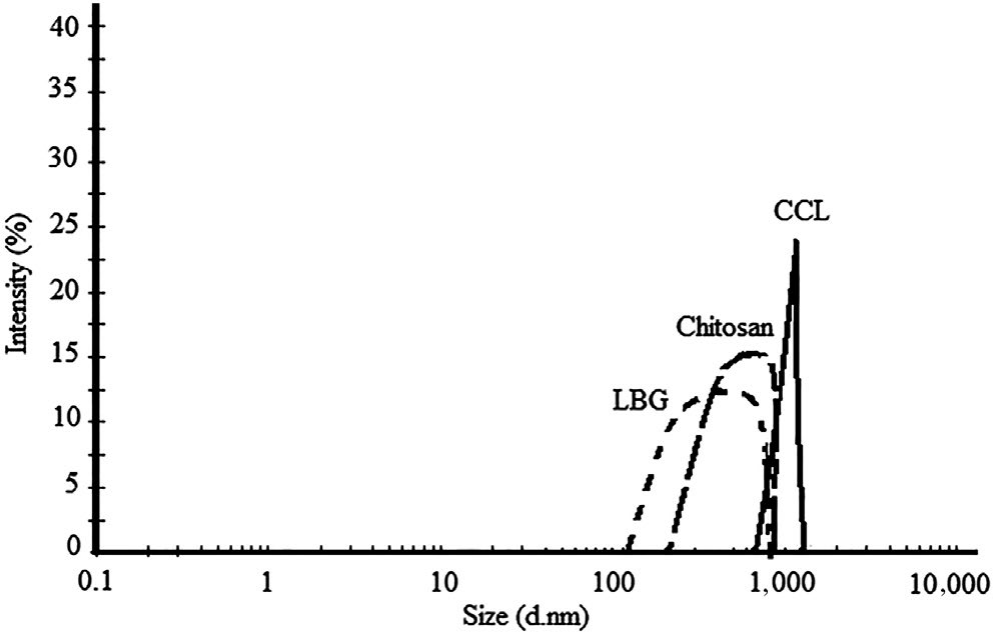
Particle size distribution of W/O/W emulsions of single‐layer LBG and chitosan and two‐layer CCL. CCL, complex of chitosan, and LBG (1:1); LBG, locust bean gum

To reduce droplet size and homogeneity, a pressure of 9000–13,000 psi (11,500 psi) was used, but the pressure above 11,500 psi increased the droplet size which can be associated with re‐accumulation and consequently increasing the droplet size of emulsions (Table 1). Therefore, the pressure of 11,500 psi was used to as optimum pressure for homogenization of emulsions at 3, 5, and 7‐time cycles (90 s each cycle). Also, an increase in inhomogeneous input pressure from an optimal level resulted in an increment in the size of droplets of emulsions (Marie, Perrier‐Cornet, & Gervais, 2002). The Z‐average diameter of the W/O/W emulsions created with various coating materials was demonstrated in Table 2 while the smallest Z‐average size of the emulsion droplets created with chitosan, CCL, and LBG was observed at time cycles of 5, 7, and 5 at 115.47, 68.12, and 128.37 nm, respectively. The results showed that the combined application of chitosan and LBG was the best treatment to create multi‐emulsions. The difference between the emulsion properties of these coating materials, such as their surface activity, the rate of adsorption at the droplet surface, the ductility characteristics, and the intramolecular interactions in the oil‐water interface, can be proposed as the reasons for the difference between the Z‐average diameters of various emulsions. In a similar study, the lowest Z‐average diameter of the emulsions created by Hi‐Cap 100, a complex of whey protein isolate–basil seed gum and complex of soy protein isolate–basil seed, was measured as 318, 736.9, and 1,918 nm, respectively. It was also found that the pressure above 12,000 psi increased the Z‐average size of droplets of emulsion which can be correlated with the further deforming in the proteins’ structure during their emulsification and re‐accumulation (Delfanian et al., 2018). The dispersion curve of the droplets of emulsions prepared under high‐pressure homogenizer conditions based on the intensity parameter was shown in Figure 1. In addition to the Z‐average size, the intensity distribution is used to check the size and distribution of droplet emulsions. The results of the intensity distribution are more accurate than the Z‐average size (Delfanian et al., 2018). The curve obtained from emulsions coated with chitosan, CCL, and LBG showed a better droplet dispersion in terms of 5‐, 7‐, and 5‐time cycles in comparison with other time conditions. As each intensity curve had only one peak at 690, 872, and 338 nm, a uniform distribution of droplet size in all treatment was noted. This feature is one of the applied indicators in the determination of the stability of emulsions (Davidov‐Pardo & McClements, 2015). Therefore, in the present investigation, according to Figure 1, it was found that the emulsion produced by CCL was best due to the lower curvature and the greater uniformity of the emulsion droplet size. Moreover, based on the findings of to Delphanian et al. (2018) and Mohammadi et al. (2016), contractive results were noted which can be associated with the different behavior of the biopolymers in relation to the reduction of particle size.

**TABLE 1:** Particle size of W/O/W double emulsions stabilized by different wall materials at three‐time cycles and five pressure (psi)

Sample	Pressure	Time cycle		
		3	5	7
Chitosan	10,000	208 ± 1.8	200.3 ± 1.7	211.54 ± 1.5
	10,500	179.1 ± 1.4	172.4 ± 1.5	188.7 ± 1.8
	11,000	158.3 ± 0.2	139.6 ± 1.4	162.24 ± 1.2
	11,500	128.21 ± 0.2	115.47 ± 1.2	134.32 ± 0.5
	12,000	257.3 ± 0.1	199.22 ± 1.7	235.13 ± 0.9
CCL	10,000	175.54 ± 1.1	192.24 ± 1.5	155.62 ± 0.5
	10,500	152.33 ± 0.8	140.3 ± 1.2	126.37 ± 0.4
	11,000	93.63 ± 0.7	109.27 ± 1.1	85.4 ± 0.2
	11,500	76.61 ± 0.3	83.23 ± 1.1	68.12 ± 0.5
	12,000	105.4 ± 0.2	149.27 ± 1.3	110.24 ± 0.3
LBG	10,000	272.53 ± 0.6	205.6 ± 1.1	224.35 ± 0.3
	10,500	244.6 ± 0.3	172.18 ± 1.2	184.39 ± 0.6
	11,000	165.41 ± 0.2	150.56 ± 1.1	145.32 ± 0.5
	11,500	147.91 ± 0.4	128.37 ± 0.4	139.19 ± 1.3
	12,000	270.3 ± 0.5	266.3 ± 1.2	210.89 ± 1.4

Abbreviations: CCL, complex of chitosan and LBG (1:1); LBG, locust bean gum.

**TABLE 2:** Particle size, polydispersity index (PDI) and zeta potential of W/O/W double emulsions stabilized by different wall materials at three‐time cycles

Sample	Particle size (nm)			PDI			ζ‐potential (mV)
	3	5	7	3	5	7	
Chitosan	128.21 ± 0.2 ^b^	115.47** ± **1.2 ^a^	134.32** **± 0.5 ^b^	0.4554 ± 0.0077 ^a^	0.414 ± 0.0027 ^a^	0.505 ^a^	25.97 ± 1.1 ^a^
CCL	76.61 ± 0.3 ^c^	83.23 ± 1.1 ^c^	68.12 ± 0.5 ^c^	0.344 ± 0.0065 ^c^	0.316 ± 0.0067 ^b^	0.256 ^c^	−9.2 ± 0.4 ^b^
LBG	147.91 ± 0.4 ^a^	128.37 ± 0.4 ^b^	139.19 ± 1.3 ^a^	0.383 ± 0.0062 ^b^	0.378 ± 0.0089 ^c^	0.439 ^b^	−41.05 ± 0.9 ^c^

Note

Means ± *SD* (standard deviation) within a column with the same lowercase letters is not significantly different at *p* < .05.

LBG, locust bean gum; CCL, complex of chitosan and LBG (1:1).

The PDI represents the uniformity of dispersion in the range of 0 and 1 while the PDI close to zero indicates the homogeneous particles and larger than 0.5 indicates a nonuniform particle size distribution (Lutz, Aserin, Wicker, & Garti, 2009). The results of Table 2 showed that the PDI of nanoemulsions in all conditions was <0.5. Also, the nanoemulsions produced by CCL were the most homogeneous treatments. Additionally, in another investigation, Delphanian et al. (2018) reported that PDI levels of multiple emulsions created by various coating materials were <0.3.

ζ‐potential such as size distribution and PDI are important factors for evaluating colloidal dispersion (Delfanian et al., 2018). Colloidal systems have a surface charge during the ionization of functional groups of components or by adsorption of ions. The electrostatic repulsion produced by the surface charge of the nanoparticles prevents the accumulation of particles and, as a result, stabilizes the system. ζ‐potential is a surface charge index that controls the interaction between droplets and provides stability throughout the system (Rao & McClements, 2012). The amount of ζ‐potential of emulsions covered with chitosan, CCL, and LBG was 26, −9.2, and −41.1 mV, respectively (Table 2). If the ζ‐potential of droplets of emulsions is between −30 and 30+, it will have sufficient stability at long times (Laouini, Jaafar‐Maalej, Sfar, Charcosset, & Fessi, 2011). Therefore, it can be predicted that droplets of emulsions created with chitosan and CCL are more stable than LBG‐coating emulsions. In another study, the ζ‐potentiality was determined between −13.1 and −30.3 mV, respectively, for the emulsions covered with different wall materials (Delfanian et al., 2018).

### 3.2 Encapsulation efficiency and release properties

Encapsulation of antioxidant compounds among W/O/W double‐walled nanoemulsions has a great influence on their antioxidant activity (Li, Jiang, Xu, & Gu, 2015).

**FIGURE 2:**
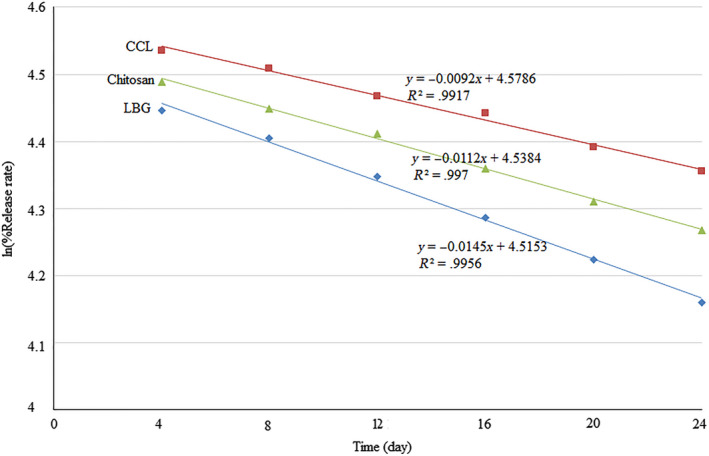
Release rate and regression equations of phenolic compounds of nanocapsules coated with LBG, chitosan, and CCL. CCL, complex of chitosan and LBG (1:1); LBG, locust bean gum

In the current investigation, the encapsulation efficiency and polyphenols release were determined for the internal aqueous phase of the double‐layer emulsions for 24 days at 30°C. At zero point, the encapsulation efficiency of the encapsulated powders produced by chitosan, CCL, and LBG was 85.3, 93.3, and 89%, respectively (Table 3). In another study, the highest initial encapsulation efficacy was associated with the encapsulated powder produced by Hi‐Cap 100 (95.25%), followed by encapsulated powders with complex of whey protein isolate–basil seed gum and complex of soybean protein isolate–basil seed gum (90.9% and 92.88% respectively) (Delfanian et al., 2018). Also, the results of 24 days of storage at 30°C showed that the least amount of encapsulation efficiency was associated with CCL, followed by LBG and chitosan (Table 3). In this regard, a significant correlation between reducing the size of droplets of emulsions and increasing the encapsulation efficiency of encapsulated powders was noted which means that the smaller size of droplets makes them more stable and consequently higher encapsulation efficiency was achieved (Salvia‐Trujillo, Decker, & McClements, 2016). The highest encapsulation efficiency was observed in CCL‐encapsulated powder with the lowest Z‐average size emulsions. In Table 3 and Figure 2, the rate constant (*k*) and half‐life period (*t*_1/2_) have been reported for encapsulated powders. The lowest rate constant (0.0092) and the highest half‐life period (75.3 days) among the various coatings related to CCL during 24 days. In the same study, the two parameters mentioned resulted by using of Hi‐Cap 100, a complex of whey protein isolate–basil seed gum, were determined to be 0.0529 and 0.0563 and 13.1 and 12.3 days, respectively (Delfanian et al., 2018).

**TABLE 3:** Encapsulation efficiency, regression analysis, and half‐life values of encapsulated powders produced with different wall materials during 40 days storage at 30°

Sample	Storage time (day)						Parameters		
	4	8	12	16	20	24	*K* (day^−1)^	*t*_1/2_ (day)	*R* ^2^
Chitosan	85.3 ± 1.1^c^	81.9 ± 0.6^c^	77.3 ± 0.5^c^	72.7 ± 0.6^c^	68.3 ± 0.6^c^	64.1 ± 1.1^c^	0.0112	61.9	.997
CCL	93.3 ± 0.6^a^	90.9 ± 0.8^a^	87.2 ± 1^a^	85 ± 1^a^	80.7 ± 0.7^a^	77.9 ± 1.2^a^	0.0092	75.3	.9917
LBG	89 ± 1.1^b^	85.6 ± 0.5^b^	82.4 ± 0.5^b^	78.3 ± 0.6^b^	74.5 ± 0.4^b^	71.4 ± 1.6^b^	0.0145	47.8	.9956

Note

Means ± *SD* (standard deviation) within a column with the same lowercase letters is not significantly different at *p* < .05.

Abbreviations: CCL, complex of chitosan and LBG (1:1); LBG, locust bean gum.

### 3.3 Effect of nanoencapsulation extracts on resistance to oxidation of soybean oil

The oil samples were mainly heat‐treated under oxidative exacerbations, and finally, with the oxidative stability analysis, the strength of the antioxidant compounds can be analyzed (Dunford, 2015). In this study, peroxide and p‐anisidine tests were used to investigate oxidative stability.

#### 3.3.1 Peroxide value

Table 4 shows the effect of the *F. persica* extract (FPE) on the oxidative stability of soybean oil compared with TBHQ. The amount of hydroperoxides, during the oxidation process, initially increased and then reduced which can be correlated with the breakdown of hydroperoxides into compounds such as aldehydes, ketones, and alcohols (Dunford, 2015). No significant difference between the amounts of initial peroxide value of all oil samples was noted while an increasing trend in all samples during 24 days of storage was observed. The highest increase was found in the control sample (686%), followed by soybean oil containing TBHQ, 100 ppm nanoencapsulated FPE with chitosan, 100 ppm free FPE, 100 ppm nanoencapsulated FPE with LBG, 200 ppm free FPE, 100 ppm nanoencapsulated FPE with CCL, 200 ppm nanoencapsulated FPE with LBG, 300 ppm free FPE, 300 ppm nanoencapsulated FPE with chitosan, 200 ppm nanoencapsulated FPE with CCL, 200 ppm nanoencapsulated FPE with chitosan, 300 ppm nanoencapsulated FPE with LBG, 300 ppm nanoencapsulated FPE with CCL where the amount of their increase in peroxide value was 601, 379, 545, 514, 501, 443, 410, 403, 396, 379, 355, 352, and 324%, respectively. It was also determined that the nanoencapsulation process increased the antioxidant strength of the FPE extract, especially with concentrations of 200 and 300 ppm while the application of CCL in the coatings had the best effect on the antioxidant activity of the nanoencapsulated extract, followed by LBG and chitosan coatings. In a study regarding the effects of nanocapsulation on the antioxidant activity of the phenolic extract of *P. atlantica* hull, this extract in nanoencapsulated form showed an antioxidant effect while compared with the free extract. The antioxidant effect of these extracts was weaker than TBHQ (Delfanian et al., 2018) which can be attributed to the type of phenolic compounds in the extracts.

**TABLE 4:** Effect of adding *Ferula persica* extract prepared by ultrasound device and water‐ethanol (FPU) and nanoencapsulated FPU produced by chitosan, CCL, and LBG at 100, 200, and 300 ppm and TBHQ (100 ppm) on peroxide value of soybean oil under accelerated storage at 60°C for day

Time (day)	peroxide value						
	Control	Free FPU			Nanoencapsulated FPU (100 ppm) produced by		
		100 ppm	200 ppm	300 ppm	chitosan	CCL	LBG
0	1.43 ± 0.05 ^a^	1.47 ± 0.05 ^a^	1.45 ± 0.05 ^a^	1.47 ± 0.05 ^a^	1.4 ± 0.02 ^a^	1.48 ± 0.02 ^a^	1.45 ± 0.03 ^a^
4	4.87 ± 0.05 ^a^	4.71 ± 0.09 ^b^	4.59 ± 0.04 ^c^	4.4 ± 0.03 ^e^	4.8 ± 0.04 ^ab^	4.49 ± 0.05 ^d^	4.6 ± 0.05 ^bc^
8	5.57 ± 0.17 ^b^	5.26 ± 0.12 ^c^	5.14 ± 0.07 ^d^	4.86 ± 0.07 ^f^	6 ± 0.07 ^a^	5 ± 0.07 ^e^	5.5 ± 0.1 ^b^
12	7.21 ± 0.4 ^a^	6.69 ± 0.34 ^ab^	6.44 ± 0.29 ^bc^	6.01 ± 0.27 ^cd^	6.8 ± 0.06 ^a^	6.22 ± 0.07 ^c^	6.3 ± 0.08 ^c^
16	8.33 ± 0.28 ^a^	7.33 ± 0.16 ^c^	6.87 ± 0.13 ^d^	6.09 ± 0.13 ^f^	7.4 ± 0.07 ^c^	6.47 ± 0.13 ^e^	6.8 ± 0.07 ^d^
20	9.12 ± 0.24 ^a^	7.98 ± 0.21 ^c^	7.5 ± 0.18 ^d^	6.68 ± 0.16 ^f^	8.2 ± 0.05	7.07 ± 0.17 ^e^	7.8 ± 0.08 ^c^
24	11.24 ± 0.1 ^a^	9.49 ± 0.11 ^c^	8.72 ± 0.08 ^d^	7.4 ± 0.07 ^f^	9.4 ± 0.07 ^c^	8.04 ± 0.08 ^e^	8.9 ± 0.12 ^d^

Time (day)	Nanoencapsulated FPU (200 ppm) produced by			Nanoencapsulated FPU (300 ppm) produced by			TBHQ (100 ppm)
	chitosan	CCL	LBG	chitosan	CCL	LBG	
0	1.49 ± 0.07 ^a^	1.44 ± 0.05 ^a^	1.45 ± 0.05 ^a^	1.43 ± 0.04	1.47 ± 0.04 ^a^	1.46 ± 0.04 ^a^	1.47 ± 0.04 ^a^
4	4.32 ± 0.05 ^f^	4.5 ± 0.03 ^d^	4.8 ± 0.09 ^ab^	4.7 ± 0.07 ^b^	4.23 ± 0.05 ^f^	4.3 ± 0.05 ^f^	4.78 ± 0.05 ^ab^
8	4.72 ± 0.07 ^gh^	5 ± 0.07 ^e^	5.4 ± 0.12 ^bc^	4.8 ± 0.08 ^fg^	4.63 ± 0.05 ^hr^	4.6 ± 0.06 ^hr^	5.4 ± 0.16 ^bc^
12	5.8 ± 0.25 ^d^	6 ± 0.27 ^cd^	6.3 ± 0.33 ^c^	6.3 ± 0.07 ^c^	5.6 ± 0.14 ^de^	5.57 ± 0.08 ^de^	6.9 ± 0.35 ^ab^
16	5.74 ± 0.07 ^g^	6.2 ± 0.1 ^f^	6.8 ± 0.16 ^d^	6.5 ± 0.09 ^e^	5.4 ± 0.08 ^hr^	5.7 ± 0.07 ^g^	7.8 ± 0.2 ^b^
20	6.34 ± 0.14 ^g^	6.8 ± 0.16 ^f^	7.1 ± 0.21 ^e^	6.6 ± 0.07 ^f^	5.97 ± 0.14 ^hr^	6.2 ± 0.05 ^g^	8.5 ± 0.21 ^b^
24	6.78 ± 0.04 ^j^	6.9 ± 0.07 ^i^	7.4 ± 0.11 ^g^	7.1 ± 0.09 ^hr^	6.24±0.04 ^L^	6.6 ± 0.08 ^k^	10.31 ± 0.09 ^b^

Note

Means ± *SD* (standard deviation) within a row with the same lowercase letters is not significantly different at *p* < .05.

Abbreviations: CCL, complex of chitosan and LBG (1:1); LBG, locust bean gum.

#### 3.3.2 p‐anisidine value

In order to investigate the secondary oxidation, a test such as anisidine value, which is an indicator of the oxidation development and the production of secondary products of this reaction, is necessary (Dunford, 2015). Table 5 shows that the p‐anisidine value of different oil treatments for 24 days at 60°C. The amount of this index was between 1.42 and 1.44 at zero moments, with no significant difference between them. Also, the study of p‐anisidine value changes over time showed that the highest increase in this factor was observed among different treatments in pure soybean oil (463%), and the remaining samples, unlike peroxide, showed a slight increase in secondary compounds (between 31% and 44%) during the maintenance period. Therefore, it was found that the effect of free and nanocapsulated FPE on preventing an increase in p‐anisidine value as a secondary oxidation index was much higher than their effect on peroxide value (initial oxidation index). In another investigation, unlike the present study, nanoencapsulation of 100, 200, and 300 ppm of phenolic extract of *P. atlantica* hull extract using combined coatings of whey protein isolate–basil seed gum and their combination reduced the secondary oxidation of soybean oil while compared to the free extract (Delfanian et al., 2018).

**TABLE 5:** Effect of adding *Ferula persica* extract prepared by ultrasound device and water–ethanol (FPU) and nanoencapsulated FPU produced by chitosan, CCL, and LBG at 100, 200, and 300 ppm and TBHQ (100 ppm) on p‐anisidine value of soybean oil under accelerated storage at 60°C for days

Time (Day)	carbonyl value						
	Control	Free FPU			Nanoencapsulated FPU (100 ppm) produced by		
		100 ppm	200 ppm	300 ppm	chitosan	CCL	LBG
0	1.44 ± 0.06 ^a^	1.44 ± 0.06 ^a^	1.44 ± 0.06 ^a^	1.43 ± 0.03 ^a^	1.43 ± 0.04 ^a^	1.44 ± 0.05 ^a^	1.44 ± 0.05 ^a^
4	2.5 ± 0.08 ^a^	1.39 ± 0.05 ^b^	1.36 ± 0.06 ^bc^	1.25 ± 0.04^cd^	1.36 ± 0.04 ^bc^	1.34 ± 0.04 ^bc^	1.35 ± 0.04 ^bc^
8	3.54 ± 0.11 ^a^	1.47 ± 0.08 ^bc^	1.44 ± 0.07 ^bc^	1.39 ± 0.05 ^c^	1.44 ± 0.05 ^bc^	1.42 ± 0.07 ^bc^	1.42 ± 0.05 ^bc^
12	4.72 ± 0.08 ^a^	1.62 ± 0.03 ^b^	1.57 ± 0.03 ^bc^	1.49 ± 0.02 ^d^	1.51 ± 0.06 ^cd^	1.5 ± 0.02 ^d^	1.5 ± 0.04 ^cd^
16	6.79 ± 0.06 ^a^	1.67 ± 0.05 ^b^	1.62 ± 0.07 ^bc^	1.53 ± 0.07	1.6 ± 0.07 ^bc^	1.56 ± 0.07 ^c^	1.58 ± 0.02 ^c^
20	7.91 ± 0.07 ^a^	1.81 ± 0.08 ^bc^	1.75 ± 0.09 ^bc^	1.73 ± 0.1 ^bc^	1.75 ± 0.06 ^bc^	1.69 ± 0.07 ^cd^	1.72 ± 0.05 ^cd^
24	8.11 ± 0.06 ^a^	2.04 ± 0.04 ^b^	2.01 ± 0.03 ^bc^	1.92 ± 0.04^de^	2.02 ± 0.04 ^b^	1.99 ± 0.04 ^bc^	2 ± 0.06 ^bc^

Time (day)	Nanoencapsulated FPU (200 ppm) produced by			Nanoencapsulated FPU (300 ppm) produced by			TBHQ (100 ppm)
	Chitosan	CCL	LBG	chitosan	CCL	LBG	
0	1.44 ± 0.03 ^a^	1.44 ± 0.05 ^a^	1.43 ± 0.02 ^a^	1.42 ± 0.01 ^a^	1.4 ± 0.02 ^a^	1.43 ± 0.02 ^a^	1.44 ± 0.06 ^a^
4	1.35 ± 0.05 ^b^	1.23 ± 0.04 ^d^	1.29 ± 0.03 ^c^	1.29 ± 0.02 ^c^	1.22 ± 0.03 ^d^	1.27 ± 0.04 ^cd^	1.44 ± 0.07 ^b^
8	1.42 ± 0.06 ^bc^	1.41 ± 0.03 ^bc^	1.41 ± 0.04 ^bc^	1.47 ± 0.03 ^b^	1.39 ± 0.03 ^c^	1.48 ± 0.03 ^b^	1.53 ± 0.11 ^b^
12	1.6 ± 0.02 ^b^	1.51 ± 0.03 ^cd^	1.54 ± 0.03 ^cd^	1.58 ± 0.03 ^b^	1.48 ± 0.05 ^d^	1.55 ± 0.02 ^c^	1.66 ± 0.05 ^b^
16	1.57 ± 0.03 ^c^	1.51 ± 0.04 ^d^	1.53 ± 0.04 ^cd^	1.57 ± 0.04 ^c^	1.48 ± 0.06 ^d^	1.53 ± 0.03 ^cd^	1.73 ± 0.07 ^b^
20	1.72 ± 0.04 ^cd^	1.66 ± 0.07 ^cd^	1.68 ± 0.02 ^cd^	1.74 ± 0.04 ^b^	1.63 ± 0.09 ^d^	1.68 ± 0.04 ^cd^	1.86 ± 0.08 ^b^
24	1.95 ± 0.04 ^cd^	1.88 ± 0.03^e^	1.9 ± 0.02^de^	1.92 ± 0.03^de^	1.87 ± 0.06^de^	1.89 ± 0.05 ^de^	2.08 ± 0.05 ^b^

Note

Means ± *SD* (standard deviation) within a row with the same lowercase letters is not significantly different at *p* < .05.

Abbreviations: CCL, complex of chitosan and LBG (1:1); LBG, locust bean gum.

### 3.4 Phenolic compounds release

**FIGURE 3:**
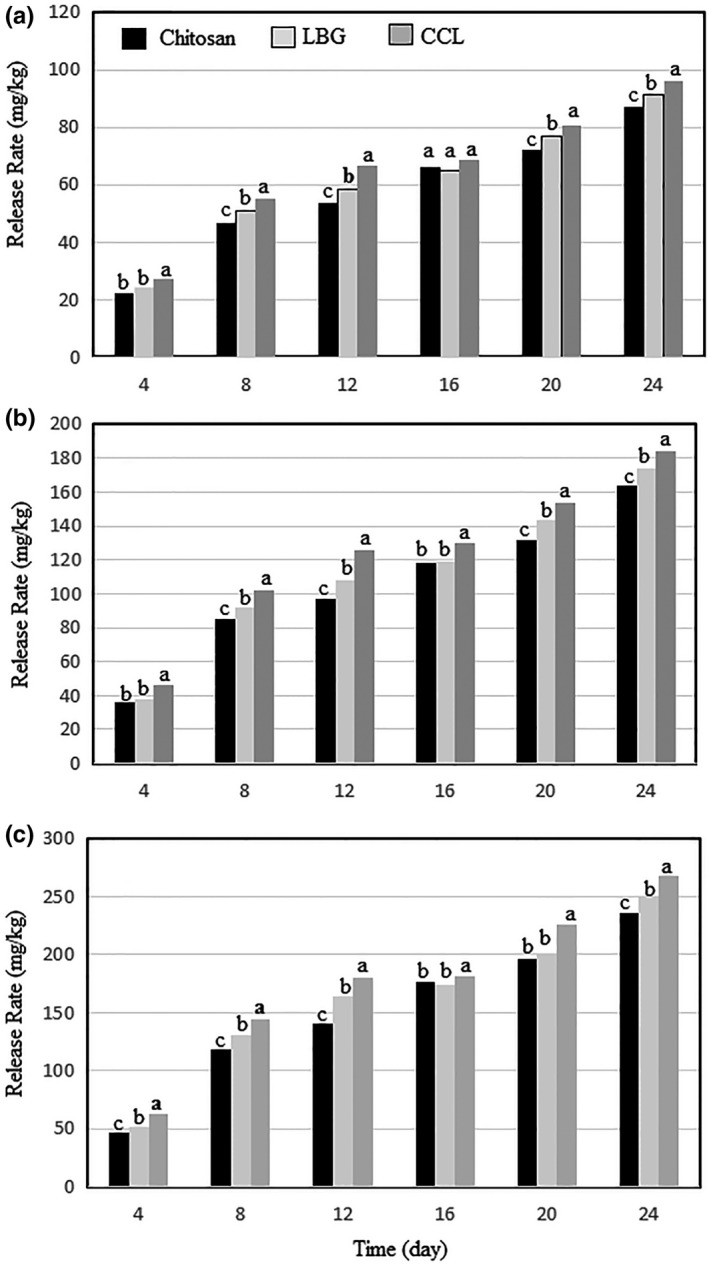
The release rate of phenolic compounds in different oil samples from encapsulated powders produced by LBG, chitosan, and CCL at levels of 100 (a), 200 (b), and 300 (c). CCL, complex of chitosan and LBG (1:1); LBG, locust bean gum

The results of peroxide and p‐anisidine value tests showed that the use of coating materials to encapsulation increased their antioxidant effect compared with TBHQ and FPE. Therefore, in order to better identify their function, the release of phenolic compounds from extracts encapsulated into soybean oil was investigated during storage at 60°C for 24 days by measuring total phenolic compounds (Figure 3). By increasing the concentration of nanoencapsulated phenolic compounds (FPE), the release rate of these compounds decreased during 24 days. It was also observed that the highest phenolic compounds were released in soybean oil containing FPE nanocapsulated with CCL, followed by FPE samples with LBG and chitosan. The results of the peroxide number test showed that CCL had the best oxidative stability in soybean oil, and then LBG and chitosan respectively, which were consistent with the results of phenolic compounds release. Also, the encapsulation of phenolic compounds from some medicinal plants using the alginate–chitosan system can increase the antioxidant power of these compounds while compared with the free extract (Belščak‐Cvitanović et al., 2011). Additionally, the amount of phenolic compounds released from the nanoencapsulated extract of *P. atlantica* hull among whey protein isolate–basil seed gum and soy protein isolate–basil seed samples cannot delay the oxidation of soybean oil (Delfanian et al., 2018).

## 4 CONCLUSION

In the present study, the first, the *F. persica* extract (FPE) was used to make nanoemulsions with different coatings. The evaluating the nanoemulsions coated with chitosan, CCL, and LBG showed that using CCL(a combination of LBG and chitosan 1:1) produced the best W/O/W nanoemulsion and followed by LBG and chitosan, respectively. Also, to investigate the effect of nanocapsulated extracts on oxidative stability of soybean oil, changes of peroxide value and p‐anisidine value were measured at 60°C for 24 days. While the results of the peroxide value test showed that the nanoencapsulation of the FPE with deferent coating materials caused the positive effect on resistance to oxidation of soybean oil while compared with TBHQ and free FPE, although in the p‐anisidine value test, there was not found a significant difference between the effects of different treatments in soybean oil. Between deferent coatings, CCL had the best coverage caused by the release of more phenolic compounds than the LBG and chitosan.

## CONFLICTS OF INTEREST

The authors declare no conflict of interest.

## ETHICAL APPROVAL

This article does not contain any studies with human participants or animals performed by any of the authors.
